# MET expression and copy number heterogeneity in nonsquamous non-small cell lung cancer (nsNSCLC)

**DOI:** 10.18632/oncotarget.3976

**Published:** 2015-05-15

**Authors:** David Casadevall, Javier Gimeno, Sergi Clavé, Álvaro Taus, Lara Pijuan, Miriam Arumí, Marta Lorenzo, Silvia Menéndez, Israel Cañadas, Joan Albanell, Sergio Serrano, Blanca Espinet, Marta Salido, Edurne Arriola

**Affiliations:** ^1^ Servei d'Oncologia Mèdica, Hospital del Mar, Barcelona, Spain; ^2^ Servei de Patologia, Hospital del Mar, Barcelona, Spain; ^3^ Laboratori de Citogenètica Molecular, Servei de Patologia, Hospital del Mar, Barcelona, Spain; ^4^ Cancer Research Program, IMIM (Hospital del Mar Medical Research Institute), Barcelona, Spain; ^5^ Universitat Pompeu Fabra, Barcelona, Spain; ^6^ Universitat Autònoma de Barcelona, Bellaterra, Spain; ^7^ Current address: Cancer Sciences Unit, University of Southampton, Southampton, UK; ^#^ Cancer Sciences Unit, University of Southampton, Southampton, UK

**Keywords:** c-MET, immunohistochemistry, FISH, non-small-cell lung cancer, heterogeneity

## Abstract

**Objective:**

We aimed to assess MET intratumoral heterogeneity and its potential impact on biomarker-based patient selection as well as potential surrogate biomarkers of MET activation.

**Methods:**

Our study included 120 patients with non-squamous Non-small-cell Lung Cancer (nsNSCLC), of which 47 were incorporated in tissue microarrays (TMA). Four morphologically distinct tumor areas were selected to assess MET heterogeneity. MET positivity by immunohistochemistry (IHC) was defined as an above-median H-score and by +2/+3 staining intensity in >50% of tumor cells (Metmab criteria). *MET* FISH positivity was defined by *MET*/CEP7 ratio ≥ 2.0 and/or *MET* ≥ 5.0. MET staining pattern (cytoplasmic vs. membranous) and mesenchymal markers were investigated as surrogates of MET activation.

**Results:**

Median MET H-score was 140 (range 0–400) and 47.8% of patients were MET positive by Metmab criteria. Eight cases (6.8%) were *MET* FISH positive and showed higher H-scores (*p* = 0.021). MET positivity by IHC changed in up to 40% of cases among different tumor areas, and *MET* amplification in 25–50%. Cytoplasmic MET staining and positivity for vimentin predicted poor survival (*p* = 0.042 and 0.047, respectively).

**Conclusions:**

MET status is highly heterogeneous among different nsNSCLC tumor areas, hindering adequate patient selection for MET-targeted therapies. MET cytoplasmic staining and vimentin might represent surrogate markers for MET activation.

## INTRODUCTION

Despite significant advances in diagnosis and treatment, lung cancer remains the leading cause of cancer death worldwide [1]. Non-small cell lung cancer (NSCLC) accounts for up to 85% of lung cancers, of which 40% are adenocarcinomas [2]. During the last decade, considerable progress has been made in the knowledge of NSCLC biology. Several molecular alterations, such as mutations in the epidermal growth factor receptor (*EGFR*) [3] or anaplastic lymphoma kinase (*ALK*) and ROS proto-oncogene 1 (*ROS1*) rearrangements [4]predict response to specific targeted therapies. These developments have greatly impacted on patients' outcome and quality of life [5–7].

*MET* was first identified in the late ‘80s, it is located on chromosome band 7q31 and encodes a heterodimeric transmembrane receptor with tyrosine kinase activity (RTK) [8, 9]. Activation of MET initiates a cascade of cellular signaling processes that ultimately lead to proliferation, reduced apoptosis, epithelial to mesenchymal transition (EMT) and an increased invasiveness and metastatic potential [10, 11]. MET pathway activation has been explained by different mechanisms such as genetic point mutations, gene amplification, post-translational activation, as well as in a ligand-dependent manner [12, 13].

The presence of MET protein overexpression and *MET* gene amplification in NSCLC are globally considered as adverse prognostic factors [14–17]. Consequently, many efforts have been made to develop MET-targeted agents [18, 19]. Clinical benefit was initially reported in patients with high serum levels of circulating HGF [20]or whose tumors harbored *MET* gene amplification [21]. In the MARQUEE [22] and the MetLung trials [23], patients were selected based on non-squamous histology and on MET immunohistochemical expression, respectively. Both trials failed to meet their primary endpoints, highlighting the need for predictive biomarkers for Met-directed treatment.

During the past few years, next-generation sequencing studies have revealed remarkable genetic and phenotypic differences among individual solid tumors [24] and also among different tumor areas and their metastases [25, 26]. This heterogeneity can interfere with biomarker-based treatment decisions, particularly when these are made based on material from small tumor biopsies.

Finally, a recent report in patients with gastric adenocarcinoma has suggested that MET staining pattern can predict *MET* gene amplification [27]. Moreover, in previous experiences with SCLC patients, we have observed that total MET protein expression does not always translate pathway activation and that signaling through MET can trigger EMT [28]. Thus, we hypothesized that the presence of a mesenchymal phenotype could translate MET pathway activation.

The primary aim of this study was to evaluate the potential impact of intra-tumor heterogeneity on MET evaluation and classification using different techniques and criteria. Furthermore, we sought to assess the correlation of MET status with other pathological and molecular characteristics. Finally and with exploratory purposes, we investigated potential surrogate markers of MET activity, such as MET staining pattern and the presence of mesenchymal markers by immunohistochemistry.

## MATERIALS AND METHODS

### Study population

Criteria for patient selection were non-squamous non-small cell lung carcinoma (nsNSCLC) histology and availability of tissue for the studies. A total of 124 tumor specimens from 120 patients diagnosed of nsNSCLC at our institution between 2009 and 2013 were included. Four of the 120 patients presented two different tumors, thus providing one extra specimen each. Material was available either from surgical resections, core-needle biopsies or cytological cell-blocks. Clinical data were extracted from medical records and included age, sex, smoking history, tumor disease stage and clinical follow-up information.

### Tissue microarray construction

Based on tissue availability, 47 of the patients were selected to construct tissue microarrays (TMAs) as outlined by Kononen *et al*. [29]. First, original Hematoxylin-Eosin (H&E) stained-sections were reviewed from each patient to identify different malignant areas and benign lung tissue. A total of six tissue cores with a 2 mm of diameter were obtained from each patient, four of them containing different histological areas of the carcinoma (named A, B, C and D) and two of them containing benign lung parenchyma. Two of the 47 patients presented two different tumors, thus providing eight tumor cores each. This lead to a final number of 196 tumor cores divided into six TMAs.

### Fluorescence *in situ* hybridization

*MET* fluorescence *in situ* hybridization (FISH) evaluation was performed on unstained formalin-fixed and paraffin-embedded(FFPE) tissue sections from the whole tumor and the TMA samples, as previously described [30], using a *MET*/CEP7 probe cocktail (#06N05-020, Abbott Molecular Inc, Des Plaines, IL) according to manufacturer's instructions. A minimum of fifty non-overlapping cells with hybridization signals were examined for each case with a BX51 fluorescence microscope (Olympus, Tokyo, Japan) and using the Cytovysion software (Applied Imaging, Grand Rapids MI). Tumors with *MET*/CEP7 ratio ≥ 2.0 (named “truly amplified”) and/or *MET* ≥ 5.0 copies (named “high polysomy”) were considered *MET* FISH positive [15, 31]. *MET* gains -defined as a mean copy number ≥ 2.5 copies in at least 10% of analysed nuclei- were also recorded.

### Immunohistochemical assays

MET immunohistochemistry (IHC) evaluation was performed using anti-total c-MET (SP44) Rabbit Monoclonal as a primary antibody (#7904430, Ventana Medical Systems, Tucson, AZ) and revealed using an Anti-RbOmniMap DAB Detection Kit (#760149, Ventana Medical Systems). The staining was carried out according to the manufacturer's protocol on a Discovery XT platform (Ventana Medical Systems). The primary antibody was incubated for 60 minutes. IHC staining was evaluated by one pathologist using two different methods. The first one was an H-score, as initially described to evaluate EGFR expression [32]. Briefly, this score ranges from 0 to 400 and results from the combination of the staining intensity (0–4) and the percentage of positive tumoral cells (0–100%) in each sample. Tumor samples were considered positive if their H-score was above median. The second one was the method described by Spigel and collaborators [33], which divides tumors into two different categories: MET high for cases presenting strong MET staining (+2 or +3) in more than 50% of tumoral cells and MET low for cases not fulfilling the former criteria. Met staining pattern, i.e. predominantly membranous vs. cytoplasmic, was assessed as described elsewhere [27].

E-cadherin and vimentin as EMT immunohistochemical markers were evaluated semiquantitatively [34]. Anti-human E-cadherin (NCH-38) mouse monoclonal primary antibody (#IR059, Dako, Carpinteria, CA) and anti-Vimentin (V9) mouse monoclonal primary antibody (#IR630, Dako) were evaluated. Both were revealed using the EnVision Flex visualization system (#K8010, Dako) and carried out according to the manufacturer's protocol using DakoAutostainer Plus. E-cadherin expression was evaluated as positive or “normal” when more than 50% of tumoral cells showed either membranous or cytoplasmatic staining. Vimentin expression was evaluated as positive or “acquired” when more than 5% of tumoral cells presented strong staining. For analysis purposes, samples showing positive E-cadherin expression were considered as having an epithelial phenotype, whereas samples showing acquired Vimentin staining were considered mesenchymal.

### Statistical analysis

All 196 TMA cores were considered and analysed as individual cases to study the association between MET IHC and *MET* FISH with histopathological variables. These associations were analysed using Chi-square or two-sample *T*-tests as necessary. Heterogeneity between different cores (A, B, C and D) was assessed using Kappa agreement index for categorical variables (i.e. FISH categories) and intraclass correlation coefficient for continuous variables (i.e. MET H-score).

Survival analyses were only performed in those patients included in the TMAs as this was a more homogeneous population, being all surgically treated patients with early stage disease. Survival curves were obtained with the Kaplan-Meier method and significance of the differences in outcome was evaluated with the Cox regression test. Statistical analysis was carried out with SPSS version 13.0 (SPSS Inc., Chicago, IL). Data and statistical analysis reported are fully compliant with the REMARK guidelines [35].

## RESULTS

### Clinical and pathological characteristics of the study population

Patients' median age was 66 years, 69% were males and 52% were current smokers. Forty percent of the study population had stage I disease and 85% were adenocarcinomas (Table 1). Most of the samples showed moderate or poor histological differentiation (Grades 2–3). The predominant histological patterns in adenocarcinomas were acinar or solid with mucin production, whereas lepidic and micropapillary patterns were less common.

**Table 1:** Global study population and TMA patients' characteristics

	Global population^1^ (*n* = 120)	TMA population^2^ (*n* = 47)
Age (yr)		
Median	66	66
Range	41–92	41–80
Sex, *n* (%)		
Male	83 (69)	29 (62)
Female	37 (31)	18 (38)
Smoking status, *n* (%)		
Never smoker	20 (17)	12 (25)
Former smoker	37 (31)	14 (30)
Current smoker	63 (52)	21 (45)
Stage, *n* (%)		
I^3^	50 (40)	29 (60)
II	19 (15)	8 (16)
III	20 (17)	10 (20)
IV	35 (28)	2 (4)
Histology, *n* (%)		
Adenocarcinoma	106 (85)	44 (90)
NOS	18 (15)	5 (10)
Histological Grade^4^, *n* (%)		
1	16 (20)	12 (30)
2	33 (42)	17 (42)
3	30 (38)	11 (28)
Not assessable	27	4
*KRAS, n* (%)		
Wild-type	90 (79)	38 (83)
Mutated	24 (21)	8 (17)
Not Assessable	10	3
*EGFR, n* (%)		
Wild-type	101 (88)	38 (79)
Mutated	14 (12)	10 (21)
Not assessable	9	1
*ALK, n* (%)		
Not rearranged	106 (98)	39 (95)
Rearranged	2 (2)	2 (5)
Not assessable	16	8

1 Includes 4 patients who had two different tumors (*n* = 124 tumors).

2 Includes 2 patients who had two different tumors (*n* = 49 tumors).

3 Includes 4 stage 0 patients.

4 Only *n* = 106 adenocarcinomas. *TMA,* Tissue microarray; *NOS*, Not otherwise specified.

Mutational data was available for more than 90% of the cases. *KRAS* and *EGFR* mutations were found in 21% and 12% of the samples, respectively, whereas 2% of the cases presented *ALK* rearrangements. Patients included in the TMA study had similar characteristics, but with a higher proportion of patients with stage I disease (60%) and *EGFR* mutated cases (21%).

### *MET* FISH analysis

*MET* status by FISH was evaluable in 117 out of 124 tumors (94.4%). We found eight *MET* positive cases (6.8%; 8/117). Four of these cases exhibited a *MET*/CEP7 ratio ≥ 2 (truly amplified) and the remaining four had five or more copies of the *MET* gene (high polysomy). *MET* gain was identified in 73 cases (62.4%), being most of them polysomic for chromosome 7 (*n* = 60) (Table 2). *MET* gains were more prevalent in adenocarcinomas with a predominantly solid histological pattern (*p* = 0.011) (data not shown). Different FISH patterns are illustrated in Supplementary Figure 1.

**Table 2:** MET IHC and *MET* FISH status among biopsy (left) and TMA (right) specimens

	Global population^1^(*n* = 115)	TMA population (*n* = 49)
MET H-score		
Median	140	90
Range	0–400	0–400
Metmab score, *n* (%)		
MET high	55 (48)	17 (35)
MET low	60 (52)	32 (65)

	**Global population**^1^ **(*n* = 117)**	**TMA population (*n* = 49)**
*MET* FISH negative		
*MET* disomic	36 (30.8)	11 (23)
*MET* gain	73 (62.4)	35 (71)
*MET* FISH positive^2^		
High polysomy	4 (3.4)	2 (4)
Truly Amplified	4 (3.4)	1 (2)

1 For FISH analysis, the core with the highest gene copy number value was selected. For IHC, H-score and Metmab score was calculated using all 4 cores (see Materials and Methods).

2 FISH positivity was defined as the average number of *MET copies* ≥5 or a *MET*/CEP7 ratio ≥2. *IHC*, immunohistochemistry; *TMA,* Tissue microarray; *FISH,* fluorescence *in situ* hybridization.

### MET IHC

MET IHC was assessable in 115 out of 124 tumors (92.7%). According to MetMab criteria, 55 cases (47.8%; 55/115) were classified as MET high, and 60 cases (52.2%; 60/115) as MET low (Table 2). Median H-score was 140 (range 0–400). According to H-score, 56 tumors were classified as positive (H-score > 140) and 59 as negative (H-score ≤ 140). Comparing both scoring methods, three cases were classified differently, one case with an H-score of 140 was classified as MET high and two cases with H-scores of 160 and 180, respectively, were classified as MET low. MET membranous staining was generally coarser than cytoplasmic staining (Supplementary Figure 2).

### Heterogeneity assessment

**Figure 1:**
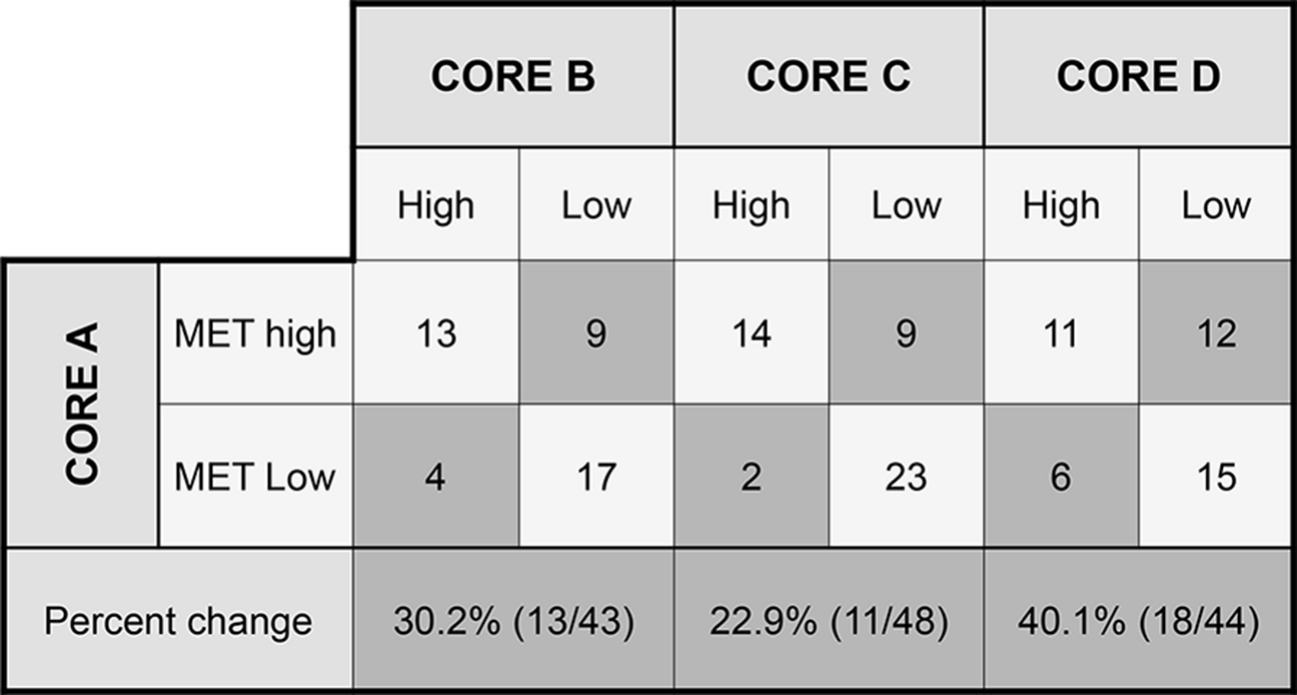
Metmab status discordance among different tumor cores Differences in MET IHC classification among different areas represented in each core. The highest variability was observed between cores A and D and the lowest between A and C.

Heterogeneity studies were focused on the TMA population, in which 171 out of 196 cores (87.2%) were assessable for histology, 176 (89.8%) for grade, 184 (93.9%) for MET IHC and 180 (91.8%) for *MET* FISH. As expected, histological pattern and grade showed a highly heterogeneous distribution among different cores (Kappa agreement index of 0.10 and 0.18, respectively, comparing A-B cores). When MET IHC status was analyzed considering the H-score as a continuous variable, intraclass correlation coefficient (ICC) was 0.47 between cores A and B. When all four cores (A to D) were included in the analysis, ICC was 0.57. When cases were classified using MetMab criteria and divided into MET high and Met low, comparison of core A with the remaining three cores (B to D) revealed differences in classification in approximately 20–40% of the cases (Figure 1).

**Figure 2:**
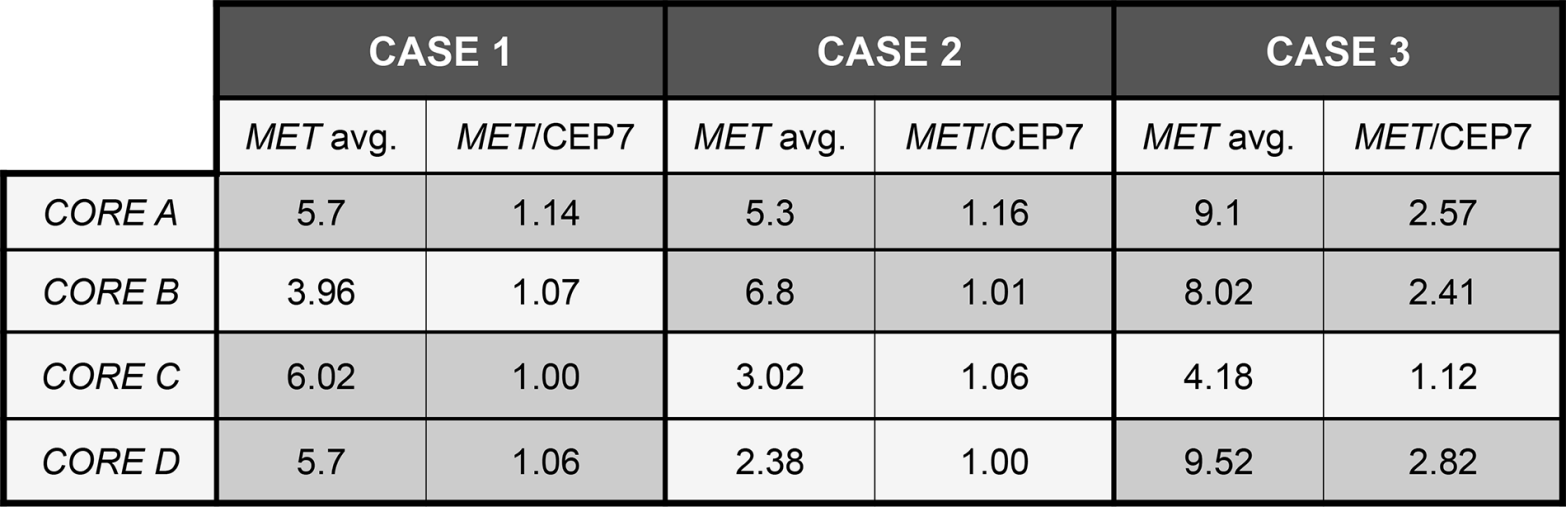
*MET* FISH discordance among different cores in FISH positive cases Eight out of twelve cores are FISH positive. None of the cases shows FISH positivity in all four cores.

**Figure 3:**
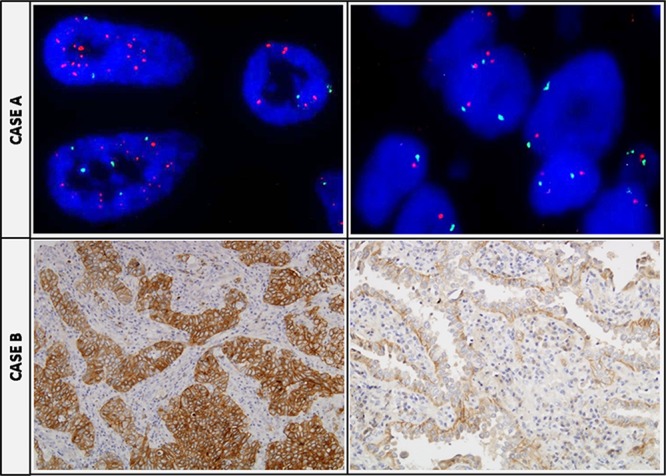
Tumor heterogeneity regarding MET status **CASE A.** Two TMA cores of the same tumor sample with opposite FISH MET results: in the left a positive core showing a MET/CEP7 ratio ≥2, and in the right a MET negative disomic case. **CASE B.** Two TMA cores of the same tumor sample with opposite MET IHC results: at the left a positive +4 area, and at the right a completely negative area of the same tumor.

Regarding *MET* FISH analysis, when evaluated as a categorical variable (*MET* disomic, *MET* gain, *MET* positive), Kappa agreement index between cores A and B was 0.35. Regarding *MET* gain as a continuous variable, ICC between the four cores was 0.58. Among the three *MET* FISH positive cases found in the TMA population, four out of the 12 cores represented were FISH negative. Moreover, none of the cases was considered positive in all four cores (Figure 2). Intra-tumor heterogeneity of MET by both IHC and FISH is illustrated in Figure 3.

### Association between IHC and FISH

**Figure 4:**
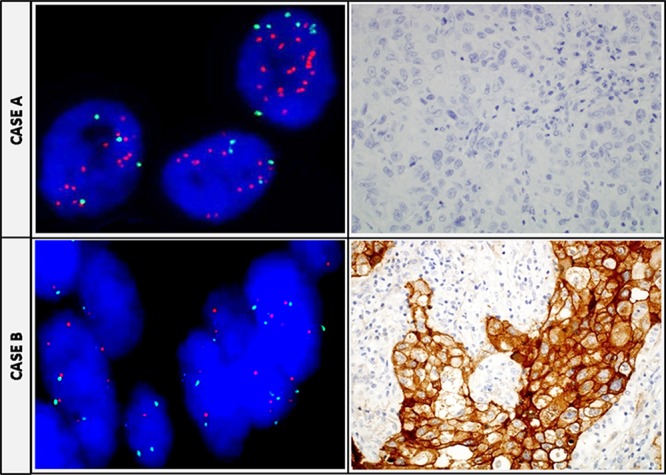
Discordance between FISH and IHC in individual tumors **CASE A.** *MET* FISH positive case showing a *MET*/CEP7 ratio ≥2 (left) and, the same case assessed by IHC showing negative staining (right). **CASE B.** *MET* FISH negative sample (left) with a high positive score by IHC (strong +4 membranous predominant staining) in the same sample (right).

*MET* FISH positive cases had higher H-score values (*p* = 0.021) (Supplementary Figure 3). Among these, the four truly amplified cases had higher H-score values than those categorized as high polysomy 7, although these difference was not statistically significant (data not shown). However, no significant association was found between *MET* mean copy number and MET H-score considered as continuous variables (Supplementary Table 1). Applying the criteria recently proposed by Camidge *et al*. [36], only the four cases categorized as truly amplified would be considered *MET* positive tumors. Of these, one case had high-level *MET* amplification (*MET*/CEP7 ratio ≥ 5) with an H-score of 400 and MET high by MetMab criteria, whereas the remaining three cases had an intermediate-level of *MET* amplification (*MET*/CEP7 ratio ≥ 2.2- < 5), of which one was classified as MET high and the remaining two as MET low by IHC. Discordance between IHC and FISH is illustrated in Figure 4.

### MET staining pattern and mesenchymal markers

**Figure 5:**
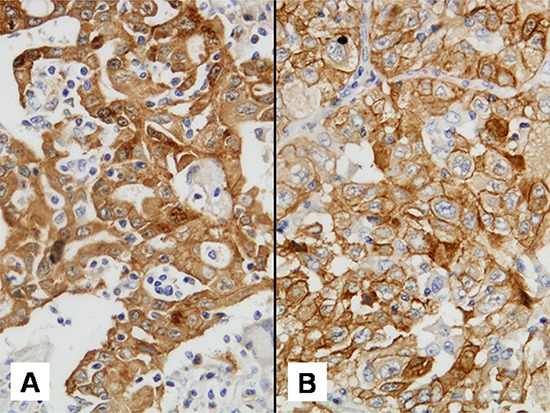
Met IHC staining pattern discordance **A.** and **B.** show different tumor cores from the same patient. A: predominantly cytoplasmic staining and B: predominantly membranous staining.

MET staining pattern was assessable in 132 cores. Out of these, only 14 (10.6%; 14/132) corresponding to 11 patients showed a predominantly cytoplasmic staining. Heterogeneity of MET staining pattern among different tumor cores was also observed (Figure 5). Three patients had predominantly cytoplasmic MET in two cores. The remaining 8 patients showed cytoplasmic staining in only one of the four cores. No patient had cytoplasmic MET in all four cores and none of the cores with cytoplasmic MET was FISH positive (Supplementary Figure 4). Interestingly, predominant cytoplasmic MET staining correlated with lower MET H-scores (*p* = 0.003), whereas non-smoking was associated with a membranous staining pattern (*p* = 0.042) (Table 3).

**Table 3:** Association of MET IHC with other histopathological features in TMA samples (*n* = 196 cores)

	MET H-score med [P_25_–P_75_]	*p*-value
Histological pattern		
Acinar	35 [0–280]	0.033
Lepidic	400 [300–400]	
Solid	30 [0–400]	
Papillary	25 [0–78.5]	
Histological grade		
1	360 [97.5–400]	0.010
2	60 [0–200]	
3	30 [0–383]	
Staining pattern		
Cytoplasmic	20 [7.25–160]	0.003
Membranous	240 [40–400]	
Vimentin		
Positive	0 [0–150]	0.027
Negative	80 [50–340]	
E-cadherin		
Positive	80 [1–350]	0.003
Negative	0 [0–20]	

*TMA,* Tissue microarray; *IHC*, immunohistochemistry

Vimentin staining was assessable in 184 cores and E-cadherin in 181. A total of 19 cores corresponding to eight different patients (10.3%; 19/184) showed a mesenchymal phenotype (strong vimentin staining). All of these patients had a smoking history (five of them were current smokers and the remaining three were former smokers). Interestingly, the presence of a mesenchymal phenotype was associated with a predominantly cytoplasmic MET staining (*p* = 0.042). Also, tumors showing mesenchymal features had significantly lower H-scores (*p* = 0.027), whereas the opposite occurred for E-cadherin positive tumors, which had significantly higher H-scores (*p* = 0.003) (Table 3).

### Survival analysis (TMA cohort)

**Figure 6:**
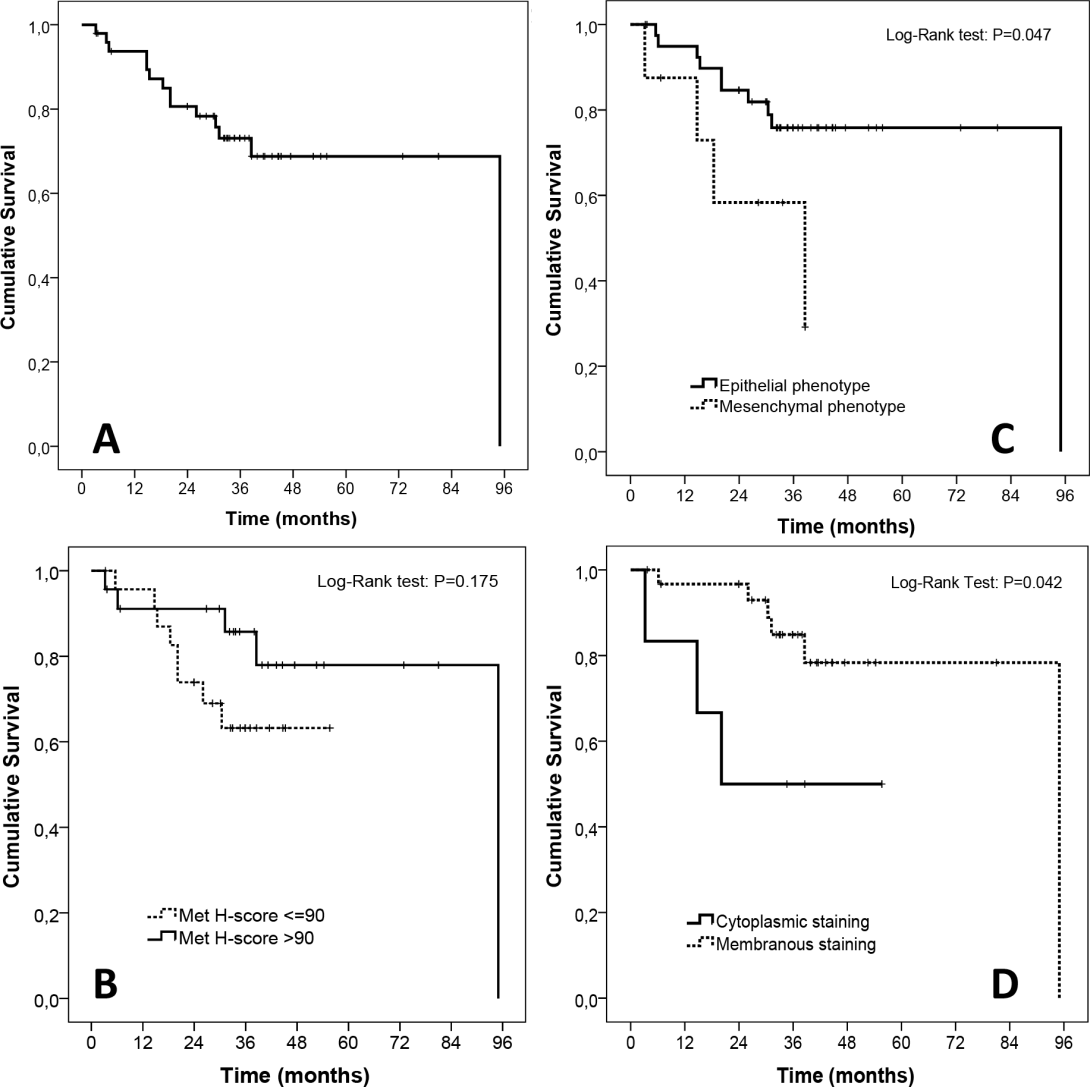
Exploratory survival analyses in the TMA cohort **A.** Overall survival of the whole cohort (*n* = 47 patients, median overall survival not reached). **B.** Survival according to MetMab status. **C.** Survival according to MET staining pattern. **D.** Survival according to epithelial or mesenchymal phenotype.

Median follow-up time was 73.2 months and median survival time was not reached. One-, two- and three-year survival rates were 93.7%, 80.6% and 73.1%, respectively. Patients whose tumor Met H-score values were below the median had shorter survival times when compared with patients with above-median values, but thiswas not statistically significant (*p* = 0.175). Interestingly, patients with tumors showing either a predominantly cytoplasmic Met staining or expression of mesenchymal features (i.e. vimentin positivity) had shorter survival times, and these differences were statistically significant (*p* = 0.042 and *p* = 0.047, respectively). Survival curves are illustrated in Figure 6.

## DISCUSSION

Lung adenocarcinoma is a morphologically heterogeneous disease. Multiple histological patterns can be identified when surgical samples are evaluated [37]. This may be due to underlying genetic heterogeneity as described for other neoplasms [38, 39] as well as for NSCLC [40, 41]. In routine clinical practice, we use biopsy or cytology samples, which contain only a small fraction of tumor, to make treatment decisions and select patients for clinical trials. In our study, patient classification by IHC could vary in up to 40% among different tumor areas.

We also corroborated that *de novo MET* amplification is a rare event, in the range of other genetic alterations such as *ROS* or *ALK* rearrangements [42]. Furthermore, although FISH positive cases showed significantly higher MET H-score values, correlation between *MET* gains and total MET protein expression was poor. We also identified cases with evident discordance between MET IHC and FISH, for which the underlying mechanism is not clear. However, it is consistent with findings from other studies [43].

The difficulty of finding the correct predictive biomarker for MET-targeted therapies may explain, at least in part, the lack of success of the two largest trials testing MET inhibitors combined with Erlotinib in NSCLC patients. The MARQUEE trial [22] selected patients with nsNSCLC histology based on data of a phase II with Tivantinib [44]. The MetLung trial [23, 33], also based on phase II data with Onartuzumab [33], performed a more restrictive selection, including only patients with +2/+3 staining in at least 50% of tumor cells. Interestingly, a molecular-based post-hoc analysis was conducted on approximately 40% of the patients (based on tissue availability) participating in the MARQUEE trial. This analysis revealed a survival benefit in those patients with high MET protein expression determined by by MetMab criteria (HR 0.7; *p* = 0.03) [45].

Another strategy for the development of MET inhibitors in NSCLC relies on patient selection based on *MET* gains or gene amplification. In the MARQUEE study, no statistically significant differences were observed in overall survival between *MET* amplified and non-amplified cases (HR 0.83; *p* = 0.34) [45]. Conversely, a subgroup analysis of the phase II study with Onartuzumab revealed a survival benefit for *EGFR* wild-type and *MET* FISH positive [15] patients receiving the combined treatment with Onartuzumab plus Erlotinib (HR 0.3; *p* = 0.06) [46]. Recently, data from a phase I/II trial with Crizotinib reported significant clinical responses in patients with *MET* amplification. Those patients with a *MET*/CEP7 ratio of ≥ 5.0 showed significantly better outcomes [36]. Although these results need to be confirmed in larger clinical trials, FISH-based criteria appear to be more adequate for patient selection. If *MET* status by FISH is less heterogeneous than MET IHC remains to be determined, as small numbers in our study (only three FISH positive cases in the TMA cohort) prevent us from drawing any robust conclusions.

Classically, it has been accepted that, after activation at the cell membrane, tyrosine-kinasereceptors (RTK) are internalized and degraded or recycled back to the membrane. However, during the last decade, preclinical evidence has emerged that highlights the role of receptor endocytosis and intracellular trafficking in RTK-mediated signaling [47–49]. In a preclinical model with immortalized bronchial cells, sustained stimulation with HGF caused a gradual displacement of c-MET receptor from the membrane to the cytoplasm [50]. Also, recent studies have associated the presence of cytoplasmic Met determined by IHC with tumor progression in patients with resected bladder cancer [51] and with poor outcome in patients with gastric adenocarcinoma [27] and mesothelioma [50]. Also, the presence of a mesenchymal phenotype, which can be an early event in NSCLC [52], has been linked to poor prognosis and metastasis development in surgically resected NSCLC [53].

Finally, our evaluation of potential surrogate markers for MET activation revealed interesting findings. Predominant cytoplasmic staining, which may translate MET pathway activation, was associated with a mesenchymal phenotype, which in turn can also be derived from MET HGF-dependent activation [34]. Although only hypothesis-generating, these results would be concordant with these patients presenting a worse prognosis, as observed in our limited series and harbor a potential predictive value for MET inhibitor benefit.

In conclusion, our study shows that MET status is highly heterogeneous within nsNSCLC tumors. This notion challenges current techniques and criteria for selecting patients for MET-targeted therapies. Further studies are needed to accurately detect patients with MET-driven tumors.

## SUPPLEMENTARY FIGURES AND TABLE



## References

[1] Jemal A, Bray F, Center MM, Ferlay J, Ward E, Forman D (2011). Global cancer statistics. CA Cancer J Clin.

[2] Herbst RS, Heymach JV, Lippman SM (2008). Lung cancer. N Engl J Med.

[3] Mok TS, Wu YL, Thongprasert S, Yang CH, Chu DT, Saijo N, Sunpaweravong P, Han B, Margono B, Ichinose Y, Nishiwaki Y, Ohe Y, Yang JJ (2009). Gefitinib or carboplatin-paclitaxel in pulmonary adenocarcinoma. N Engl J Med.

[4] Scagliotti G, Stahel RA, Rosell R, Thatcher N, Soria JC (2012). ALK translocation and crizotinib in non-small cell lung cancer: an evolving paradigm in oncology drug development. Eur J Cancer.

[5] Camidge DR, Bang YJ, Kwak EL, Iafrate AJ, Varella-Garcia M, Fox SB, Riely GJ, Solomon B, Ou SH, Kim DW, Salgia R, Fidias P, Engelman JA (2012). Activity and safety of crizotinib in patients with ALK-positive non-small-cell lung cancer: updated results from a phase 1 study. Lancet Oncol.

[6] Rosell R, Carcereny E, Gervais R, Vergnenegre A, Massuti B, Felip E, Palmero R, Garcia-Gomez R, Pallares C, Sanchez JM, Porta R, Cobo M, Garrido P (2012). Erlotinib versus standard chemotherapy as first-line treatment for European patients with advanced EGFR mutation-positive non-small-cell lung cancer (EURTAC): a multicentre, open-label, randomised phase 3 trial. Lancet Oncol.

[7] Lynch TJ, Bell DW, Sordella R, Gurubhagavatula S, Okimoto RA, Brannigan BW, Harris PL, Haserlat SM, Supko JG, Haluska FG, Louis DN, Christiani DC, Settleman J (2004). Activating mutations in the epidermal growth factor receptor underlying responsiveness of non-small-cell lung cancer to gefitinib. N Engl J Med.

[8] Park M, Dean M, Kaul K, Braun MJ, Gonda MA, Vande Woude G (1987). Sequence of MET protooncogene cDNA has features characteristic of the tyrosine kinase family of growth-factor receptors. Proc Natl Acad Sci U S A.

[9] Giordano S, Ponzetto C, Di Renzo MF, Cooper CS, Comoglio PM (1989). Tyrosine kinase receptor indistinguishable from the c-met protein. Nature.

[10] Thiery JP, Acloque H, Huang RY, Nieto MA (2009). Epithelial-mesenchymal transitions in development and disease. Cell.

[11] Birchmeier C, Birchmeier W, Gherardi E, Vande Woude GF (2003). Met, metastasis, motility and more. Nat Rev Mol Cell Biol.

[12] Gherardi E, Birchmeier W, Birchmeier C, Vande Woude G (2012). Targeting MET in cancer: rationale and progress. Nat Rev Cancer.

[13] Ma PC, Jagadeeswaran R, Jagadeesh S, Tretiakova MS, Nallasura V, Fox EA, Hansen M, Schaefer E, Naoki K, Lader A, Richards W, Sugarbaker D, Husain AN (2005). Functional expression and mutations of c-Met and its therapeutic inhibition with SU11274 and small interfering RNA in non-small cell lung cancer. Cancer Res.

[14] Takanami I, Tanana F, Hashizume T, Kikuchi K, Yamamoto Y, Yamamoto T, Kodaira S (1996). Hepatocyte growth factor and c-Met/hepatocyte growth factor receptor in pulmonary adenocarcinomas: an evaluation of their expression as prognostic markers. Oncology.

[15] Cappuzzo F, Marchetti A, Skokan M, Rossi E, Gajapathy S, Felicioni L, Del Grammastro M, Sciarrotta MG, Buttitta F, Incarbone M, Toschi L, Finocchiaro G, Destro A (2009). Increased MET gene copy number negatively affects survival of surgically resected non-small-cell lung cancer patients. J Clin Oncol.

[16] Dziadziuszko R, Wynes MW, Singh S, Asuncion BR, Ranger-Moore J, Konopa K, Rzyman W, Szostakiewicz B, Jassem J, Hirsch FR (2012). Correlation between MET gene copy number by silver *in situ* hybridization and protein expression by immunohistochemistry in non-small cell lung cancer. J Thorac Oncol.

[17] Park S, Choi YL, Sung CO, An J, Seo J, Ahn MJ, Ahn JS, Park K, Shin YK, Erkin OC, Song K, Kim J, Shim YM (2012). High MET copy number and MET overexpression: poor outcome in non-small cell lung cancer patients. Histol Histopathol.

[18] Martens T, Schmidt NO, Eckerich C, Fillbrandt R, Merchant M, Schwall R, Westphal M, Lamszus K (2006). A novel one-armed anti-c-Met antibody inhibits glioblastoma growth *in vivo*. Clin Cancer Res.

[19] Christensen JG, Burrows J, Salgia R (2005). c-Met as a target for human cancer and characterization of inhibitors for therapeutic intervention. Cancer Lett.

[20] Catenacci DV, Henderson L, Xiao SY, Patel P, Yauch RL, Hegde P, Zha J, Pandita A, Peterson A, Salgia R (2011). Durable complete response of metastatic gastric cancer with anti-Met therapy followed by resistance at recurrence. Cancer Discov.

[21] Ou SH, Bazhenova L, Camidge DR, Solomon BJ, Herman J, Kain T, Bang YJ, Kwak EL, Shaw AT, Salgia R, Maki RG, Clark JW, Wilner KD (2010). Rapid and dramatic radiographic and clinical response to an ALK inhibitor (crizotinib, PF02341066) in an ALK translocation-positive patient with non-small cell lung cancer. J Thorac Oncol.

[22] Scagliotti GV, Novello S, Schiller JH, Hirsh V, Sequist LV, Soria JC, von Pawel J, Schwartz B, Von Roemeling R, Sandler AB (2012). Rationale and design of MARQUEE: a phase III, randomized, double-blind study of tivantinib plus erlotinib versus placebo plus erlotinib in previously treated patients with locally advanced or metastatic, nonsquamous, non-small-cell lung cancer. Clin Lung Cancer.

[23] Spigel DR, Edelman MJ, Mok T, O'Byrne K, Paz-Ares L, Yu W, Rittweger K, Thurm H (2012). Treatment Rationale Study Design for the MetLung Trial: A Randomized, Double-Blind Phase III Study of Onartuzumab (MetMAb) in Combination With Erlotinib Versus Erlotinib Alone in Patients Who Have Received Standard Chemotherapy for Stage IIIB or IV Met-Positive Non-Small-Cell Lung Cancer. Clin Lung Cancer.

[24] Parsons DW, Jones S, Zhang X, Lin JC, Leary RJ, Angenendt P, Mankoo P, Carter H, Siu IM, Gallia GL, Olivi A, McLendon R, Rasheed BA (2008). An integrated genomic analysis of human glioblastoma multiforme. Science.

[25] Tao Y, Ruan J, Yeh SH, Lu X, Wang Y, Zhai W, Cai J, Ling S, Gong Q, Chong Z, Qu Z, Li Q, Liu J (2011). Rapid growth of a hepatocellular carcinoma and the driving mutations revealed by cell-population genetic analysis of whole-genome data. Proc Natl Acad Sci U S A.

[26] Gerlinger M, Quezada SA, Peggs KS, Furness AJ, Fisher R, Marafioti T, Shende VH, McGranahan N, Rowan AJ, Hazell S, Hamm D, Robins HS, Pickering L (2013). Ultra-deep T cell receptor sequencing reveals the complexity and intratumour heterogeneity of T cell clones in renal cell carcinomas. J Pathol.

[27] Ha SY, Lee J, Kang SY, Do IG, Ahn S, Park JO, Kang WK, Choi MG, Sohn TS, Bae JM, Kim S, Kim M, Kim S (2013). MET overexpression assessed by new interpretation method predicts gene amplification and poor survival in advanced gastric carcinomas. Mod Pathol.

[28] Arriola E, Canadas I, Arumi-Uria M, Domine M, Lopez-Vilarino JA, Arpi O, Salido M, Menendez S, Grande E, Hirsch FR, Serrano S, Bellosillo B, Rojo F (2011). MET phosphorylation predicts poor outcome in small cell lung carcinoma and its inhibition blocks HGF-induced effects in MET mutant cell lines. Br J Cancer.

[29] Kononen J, Bubendorf L, Kallioniemi A, Barlund M, Schraml P, Leighton S, Torhorst J, Mihatsch MJ, Sauter G, Kallioniemi OP (1998). Tissue microarrays for high-throughput molecular profiling of tumor specimens. Nat Med.

[30] Salido M, Tusquets I, Corominas JM, Suarez M, Espinet B, Corzo C, Bellet M, Fabregat X, Serrano S, Sole F (2005). Polysomy of chromosome 1 in breast cancer tumors showing an overexpression of ERBB2: a study of 15 cases using fluorescence *in situ* hybridization and immunohistochemistry. Breast Cancer Res.

[31] Camidge DR, Kono SA, Flacco A, Tan AC, Doebele RC, Zhou Q, Crino L, Franklin WA, Varella-Garcia M (2010). Optimizing the detection of lung cancer patients harboring anaplastic lymphoma kinase (ALK) gene rearrangements potentially suitable for ALK inhibitor treatment. Clin Cancer Res.

[32] Cappuzzo F, Hirsch FR, Rossi E, Bartolini S, Ceresoli GL, Bemis L, Haney J, Witta S, Danenberg K, Domenichini I, Ludovini V, Magrini E, Gregorc V (2005). Epidermal growth factor receptor gene and protein and gefitinib sensitivity in non-small-cell lung cancer. J Natl Cancer Inst.

[33] Spigel DR, Ervin TJ, Ramlau RA, Daniel DB, Goldschmidt JH, Blumenschein GR, Krzakowski MJ, Robinet G, Godbert B, Barlesi F, Govindan R, Patel T, Orlov SV (2013). Randomized phase II trial of Onartuzumab in combination with erlotinib in patients with advanced non-small-cell lung cancer. J Clin Oncol.

[34] Canadas I, Rojo F, Taus A, Arpi O, Arumi-Uria M, Pijuan L, Menendez S, Zazo S, Domine M, Salido M, Mojal S, Garcia de Herreros A, Rovira A (2014). Targeting Epithelial-to-Mesenchymal Transition with Met Inhibitors Reverts Chemoresistance in Small Cell Lung Cancer. Clin Cancer Res.

[35] McShane LM, Altman DG, Sauerbrei W, Taube SE, Gion M, Clark GM (2005). Statistics Subcommittee of the NCIEWGoCD. Reporting recommendations for tumor marker prognostic studies (REMARK). J Natl Cancer Inst.

[36] Camidge DR, Ou S-HI, Shapiro G, Otterson GA, Villaruz LC, Villalona-Calero MA, Iafrate AJ, Varella-Garcia M, Dacic S, Cardarella S, Zhao W, Tye L, Stephenson P (2014). Efficacy and safety of crizotinib in patients with advanced c-MET-amplified non-small cell lung cancer (NSCLC). ASCO Meeting Abstracts.

[37] Travis WD, Brambilla E, Riely GJ (2013). New pathologic classification of lung cancer: relevance for clinical practice and clinical trials. J Clin Oncol.

[38] Fisher R, Larkin J, Swanton C (2012). Inter and intratumour heterogeneity: a barrier to individualized medical therapy in renal cell carcinoma?. Front Oncol.

[39] Yachida S, Jones S, Bozic I, Antal T, Leary R, Fu B, Kamiyama M, Hruban RH, Eshleman JR, Nowak MA, Velculescu VE, Kinzler KW, Vogelstein B (2010). Distant metastasis occurs late during the genetic evolution of pancreatic cancer. Nature.

[40] Cancer Genome Atlas Research N (2014). Comprehensive molecular profiling of lung adenocarcinoma. Nature.

[41] Chen Z, Fillmore CM, Hammerman PS, Kim CF, Wong KK (2014). Non-small-cell lung cancers: a heterogeneous set of diseases. Nat Rev Cancer.

[42] Guo B, Cen H, Tan X, Liu W, Ke Q (2014). Prognostic value of MET gene copy number and protein expression in patients with surgically resected non-small cell lung cancer: a meta-analysis of published literatures. PLoS One.

[43] Xiu J, Feldman R, Bender RP, Salgia R (2014). Tumor biomarker evaluation of 6, 785 patients for combination treatment strategies in NSCLC. ASCO Meeting Abstracts.

[44] Sequist LV, von Pawel J, Garmey EG, Akerley WL, Brugger W, Ferrari D, Chen Y, Costa DB, Gerber DE, Orlov S, Ramlau R, Arthur S, Gorbachevsky I (2011). Randomized phase II study of erlotinib plus tivantinib versus erlotinib plus placebo in previously treated non-small-cell lung cancer. J Clin Oncol.

[45] Novello S, Scagliotti GV, Ramlau R, Barlesi F, Sandler AB, Von Roemeling R (2013). Efficacy Analysis for Molecular Subgroups in MARQUEE: a Randomized, Double-blind, Placebo-controlled, Phase 3 Trial of Tivantinib (ARQ 197) Plus Erlotinib versus Placebo plus Erlotinib in Previously Treated Patients with Locally Advanced or Metastatic, Non-squamous, Non-small Cell Lung Cancer (NSCLC). Poster presented at: World Congress on Lung Cancer.

[46] Koeppen H, Yu W, Zha J, Pandita A, Penuel E, Rangell L, Raja R, Mohan S, Patel R, Desai R, Fu L, Do A, Parab V (2014). Biomarker Analyses from a Placebo-Controlled Phase II Study Evaluating Erlotinib +/– Onartuzumab in Advanced Non-Small Cell Lung Cancer: MET Expression Levels Are Predictive of Patient Benefit. Clin Cancer Res.

[47] Joffre C, Barrow R, Menard L, Calleja V, Hart IR, Kermorgant S (2011). A direct role for Met endocytosis in tumorigenesis. Nat Cell Biol.

[48] Barrow R, Joffre C, Menard L, Kermorgant S (2014). Measuring the role for Met endosomal signaling in tumorigenesis. Methods Enzymol.

[49] von Zastrow M, Sorkin A (2007). Signaling on the endocytic pathway. Curr Opin Cell Biol.

[50] Levallet G, Vaisse-Lesteven M, Le Stang N, Ilg AG, Brochard P, Astoul P, Pairon JC, Bergot E, Zalcman G, Galateau-Salle F (2012). Plasma cell membrane localization of c-MET predicts longer survival in patients with malignant mesothelioma: a series of 15 cases from the MESOPATH Group. J Thorac Oncol.

[51] Kluth M, Reynolds K, Rink M, Chun F, Dahlem R, Fisch M, Hoppner W, Wagner W, Doh O, Terracciano L, Simon R, Sauter G, Minner S (2014). Reduced membranous MET expression is linked to bladder cancer progression. Cancer Genet.

[52] Prudkin L, Liu DD, Ozburn NC, Sun M, Behrens C, Tang X, Brown KC, Bekele BN, Moran C, Wistuba II (2009). Epithelial-to-mesenchymal transition in the development and progression of adenocarcinoma and squamous cell carcinoma of the lung. Mod Pathol.

[53] Soltermann A, Tischler V, Arbogast S, Braun J, Probst-Hensch N, Weder W, Moch H, Kristiansen G (2008). Prognostic significance of epithelial-mesenchymal and mesenchymal-epithelial transition protein expression in non-small cell lung cancer. Clin Cancer Res.

