# Supplementary material for: MET expression and copy number heterogeneity in nonsquamous non-small cell lung cancer (nsNSCLC)
Source: Oncotarget. 2015 May 15;6(18):16215–26. doi: 10.18632/oncotarget.3976 (PMC4599265; doi:10.18632/oncotarget.3976)
Supplement: Supplementary file 1 [file oncotarget-06-16215-s001.pdf]

SUPPLEMENTARY FIGURES AND TABLE

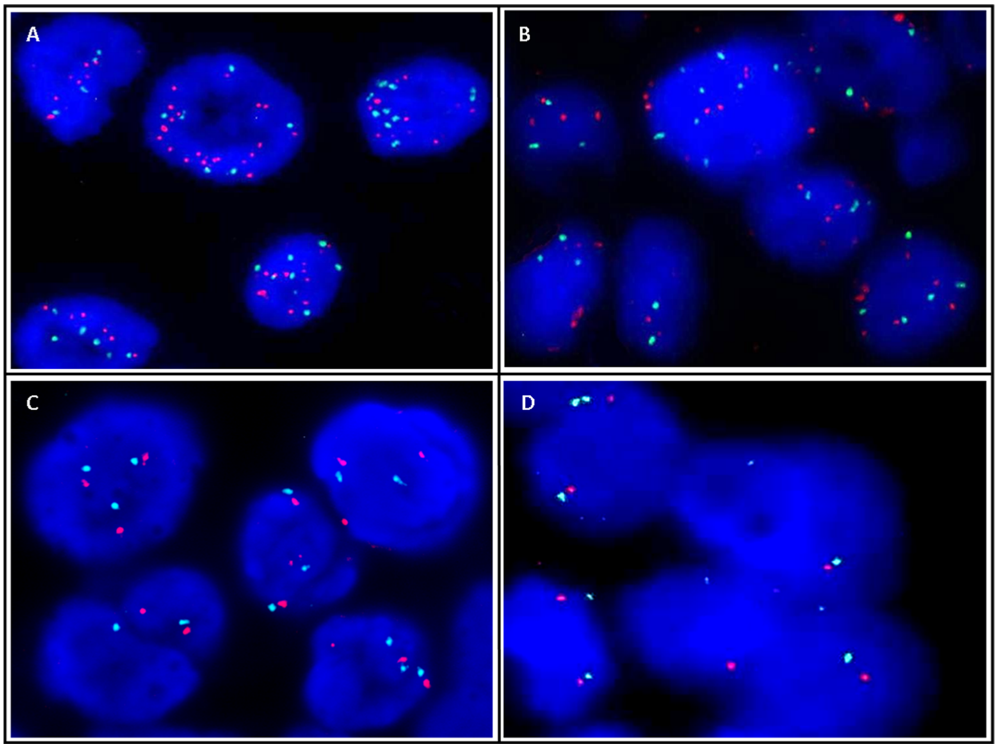

Supplementary Figure 1: Different FISH signal patterns. Four tumor samples exhibiting different *MET*/CEP7 FISH signal patterns: **A.** *MET* positive nuclei showing a *MET*/CEP7 ratio ≥ 2 (truly amplified). **B.** *MET* positive nuclei showing a *MET* ≥ 5 (high polysomy). **C.** *MET* negative nuclei showing ≥ 2.5 *MET* copies (classified as *MET* gain). **D.** *MET* negative disomic pattern.

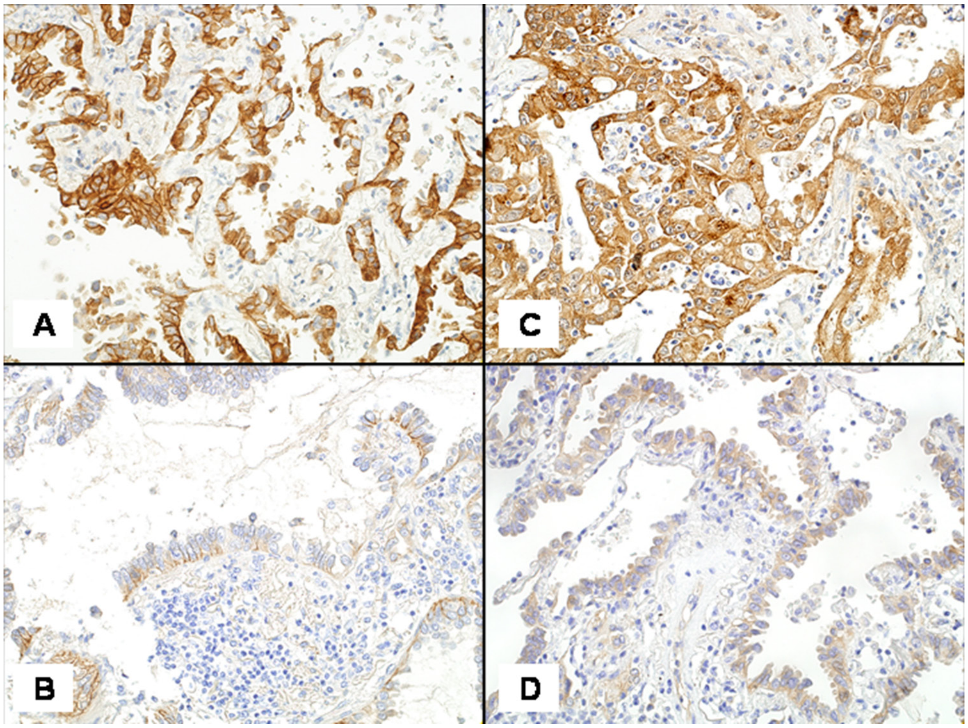

Supplementary Figure 2: Different MET staining patterns at 40x. A. strong (+4) predominantly membranous pattern (H-score: 400). **B.** weak (+1) predominantly membranous pattern (H-score: 15). **C.** strong (+4) predominantly cytoplasmic pattern (H-score: 400). **D.** weak (+1) predominantly cytoplasmic pattern (H-score: 60).

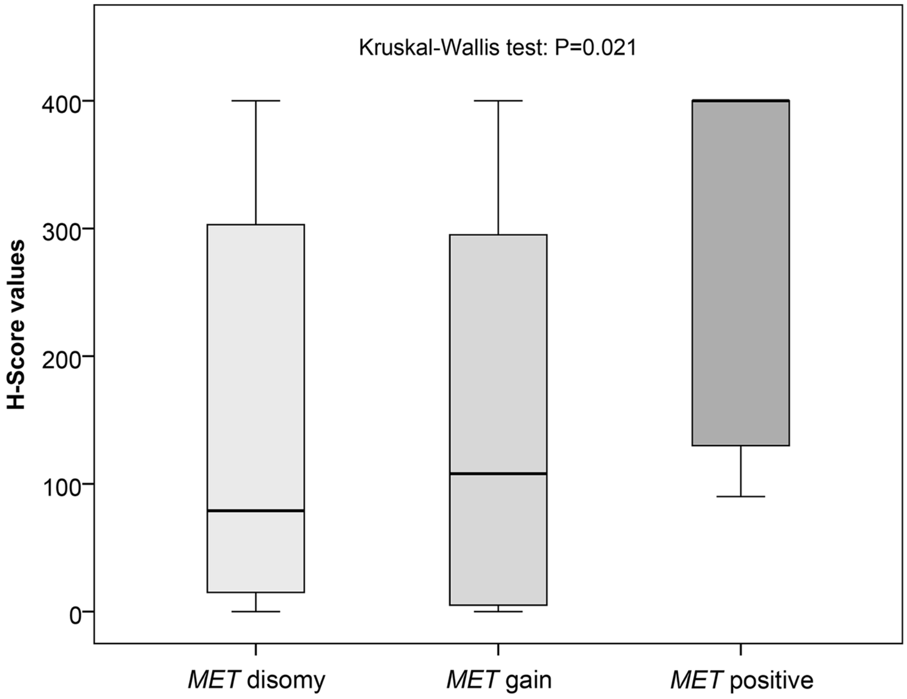

Supplementary Figure 3: MET IHC H-score differences among FISH categories. *MET* FISH positive cases (high polysomy and truly amplified) have higher MET H-scores.

		CORE B		CORE C		CORE D	
		Memb.	Cytopl.	Memb.	Cytopl.	High	Cytopl.
CORE A	Memb.	24	3	26	2	23	1
	Cytopl.	3	0	5	0	1	2
Percent change		20% (6/30)		21.2% (7/33)		0.08% (2/25)	

Supplementary Figure 4: Heterogeneity in MET IHC staining pattern among different tumor cores. Staining pattern was heterogeneous among tumor cores. No case showed cytoplasmic staining in all four cores (data not shown). Abbreviations: *Cytopl*., Cytoplasmic; *Memb*., Membranous.

Supplementary Table 1: Association between MET IHC and *MET* FISH

	MET H-score med [P_{25}–P_{75}]	*p*-value	*MET* gene copies med [P_{25}–P_{75}]	*p*-value
MET IHC				
MET high (*n* = 55)	350 [253–400]	<0.001	3 [2–4]	0.682
MET low (*n* = 60)	23 [1.38–79.75]		3 [2–3.02]	

IHC, immunohistochemistry
